# The Composition of Surgical Wound Fluids from Breast Cancer Patients is Affected by Intraoperative Radiotherapy Treatment and Depends on the Molecular Subtype of Breast Cancer

**DOI:** 10.3390/cancers12010011

**Published:** 2019-12-18

**Authors:** Katarzyna Kulcenty, Igor Piotrowski, Joanna Patrycja Wróblewska, Janusz Wasiewicz, Wiktoria Maria Suchorska

**Affiliations:** 1 Radiobiology Lab, Department of Medical Physics, Greater Poland Cancer, 61-866 Poznań, Poland; igor.piotrowski@wco.pl (I.P.); wiktoria.suchorska@wco.pl (W.M.S.); 2 Department of Electroradiology, Poznan University of Medical Sciences, ul. Garbary 15, 61-866 Poznan, Poland; 3 Department of Pathology, Poznan University of Medical Sciences and Greater Poland Cancer Center, ul. Garbary 15, 61-866 Poznan, Poland; joanna.wroblewska@wco.pl; 4 Department of Breast Cancer Surgery, Greater Poland Cancer Centre, ul. Garbary 15, 61-866 Poznań, Poland; janusz.wasiewicz@wco.pl

**Keywords:** surgery-induced inflammation, cytokines, breast cancer, breast-conserving surgery, intraoperative radiation therapy

## Abstract

Invasive oncological procedures affect the remaining tumor cells by increasing their survival, proliferation, and migration through the induction of wound healing response. The phenomena of local relapse after breast-conserving surgery (BCS) has resulted in a series of research and clinical trials with the aim of assessing whether localized intraoperative radiotherapy (IORT), may be beneficial in inhibiting local recurrences. Therefore, it is essential to assess the impact of intraoperative radiotherapy in modulating the immunological response and wound healing process. Thus, we decided to perform a quantitative analysis of the composition of surgical wound fluids (SWF) in two groups of breast cancer (BC) patients: those treated with BCS followed by IORT, and those who underwent BCS alone. We found that several cytokines, which are believed to have anti-tumor properties, were highly expressed in the luminal A breast cancer subtype in the IORT treatment group. Interestingly, we also found significant differences between IORT patients with tumors of different molecular subtypes. Based on these findings, we hypothesized that IORT treatment might be beneficial in changing the tumor bed microenvironment, making it less favorable for tumor recurrence due to decreased concentration of tumor-facilitating cytokines, especially in the luminal A subtype of BC.

## 1. Introduction

Breast cancer (BC) is the most common cancer in women. After lung cancer, it is the second most common cause of cancer-related death. Conservative breast cancer surgery followed by radiation therapy is currently the standard treatment for this type of cancer. Local recurrence is, however, of major concern after conservative breast cancer (BCS) treatment. Numerous studies have demonstrated that approximately 90% of post-surgical local recurrences occur in the same quadrant as the primary cancer. It has been shown that local recurrence originates from residual cancer cells which are present in the interstitial peritumoral tissue, peritumoral vascular spaces, or in resection margins positive for residual cancer cells. It has also been shown that peritumoral lymphatic invasion is a predictor of both local and distant metastases [1]. Breast cancer surgery of the primary tumor is required to reduce the potential of cancer cells to mutate and metastasize. However, the clinical and experimental data suggest that surgery itself may be responsible for local relapse and metastasis [2]. It has been demonstrated that invasive procedures (both surgery and biopsy) affect the remaining tumor cells, increasing their survival, proliferation, and migration [3,4]. One of the concepts explaining this phenomenon is the induction of a wound healing response. In normal tissue, such a response is necessary for tissue repair. However, induction of adaptive and innate immune response in tumor tissue stimulates cell survival, angiogenesis, and extravasation of circulating tumor cells. It has become evident that some types of immune response, or immune cells, can promote tumor progression more than others [3]. For example, tumor-resident macrophages (M2 polarized macrophages) secret vascular endothelial growth factor (VEGF) and epidermal growth factor (EGF), which induce angiogenesis and the recruitment of neutrophils contribute to tumor progression and metastasis [5,6]. Using an in vitro model, Krall et al. proved that the systemic consequences of surgery could promote the regrowth of tumor cells even at distal anatomical sites. They found out that surgery-induced tumor regrowth was associated with both local and systemic inflammatory responses characterized by the release of cytokines and the mobilization of myeloid cells into the circulation of wounded mice [7]. Several studies revealed that surgical wound fluids (SWF) derived from the surgical sites act as a stimulant factor for tumor progression. Although SWF is rich in biological factors, the expression of these factors and how they interact with tumor cells, has not been entirely explained. Agresti et al. and Wang et al. analyzed the composition of SWF from breast cancer patients in detail [8,9]. They found that the composition of SWF differs between patients depending on the treatment (quadrantectomy or mastectomy), type (invasive or in situ) and the molecular subtype of breast cancer (BC) (luminal or triple-negative). Moreover, they also found that SWF stimulated proliferation of breast cancer cells of the intrinsic subtypes in vitro. This effect was more pronounced in triple negative breast cancer (TNBC) cells than in luminal cell lines. Tagliabue et al. confirmed that higher proliferation of human epidermal growth factor receptor 2 (HER2) positive breast cancer cells occurs after stimulation with SWF [4]. Moreover, it has been demonstrated that surgery-induced inflammation promotes the stem-like phenotype and tumor-initiating abilities of breast cancer cells via STAT3 signaling [10].

The phenomenon of local relapse initiated a series of research and clinical trials aimed at assessing whether localized intraoperative radiotherapy could be as effective in inhibiting local recurrence as standard postoperative whole breast radiotherapy but better tolerated by patients in terms of shortening the therapy course. Intraoperative radiation therapy (IORT) involves the direct irradiation of diseased tissue during surgery, within the tumor bed, with doses of either 10 Gy (dose used in the Greater Poland Cancer Center, from which SWFs for the presented experiments were collected) or 23 Gy. This approach reduces the potential risk of re-population of tumor cells during the process of wound healing [11]. The studies by Belletti and Tagliabue studies showed that SWF obtained from patients who had undergone surgical removal of the breast tumor could stimulate motility and invasiveness of tumor cells in vitro. They demonstrated that wound fluid could serve as an "attractant" for residual cancer cells in the tumor bed [4,12]. Moreover, we have previously reported that SWF from patients who underwent surgery alone (BCS, WF), and those who received IORT (IORT, RT-WF) affect the stem cell phenotype in breast cancer cell lines. We also revealed reduced stimulation of the cancer stem cell (CSC) phenotype, and migratory capabilities after incubation with SWF from IORT treated patients [13,14]. Moreover, it has been shown, that SWF from IORT patients decrease the proliferation of breast cancer cells comparing to SWF after BCS alone [12]. Previously, we demonstrated that luminal (MCF7) and basal (MDA-MB-468) cell lines transit from being more epithelial to more mesenchymal cells by stimulation with BCS and IORT, and that the mesenchymal features are more pronounced after stimulation with BCS alone [14]. Our previous experiments also proved that the biological effect of SWF after IORT treatment depends on the radiation induced bystander effect (RIBE), which induces a radiobiological response in unirradiated cells [14,15]. Our results stay in compliance with the targeted intraoperative radiotherapy (TARGIT) clinical trial studies demonstrating that IORT significantly inhibits the stimulatory effects of SWF on tumor cells in vitro. This effect may be due to both cell death caused directly by ionizing radiation and to changes in the tumor microenvironment. Using proteomic analyses, Belletti et al. revealed, that TARGIT could have an antitumor effect, surpassing radiation-induced cancer cell kill through changes in the growth factors and cytokines present in the resection cavity [12]. 

Thereafter, it is important to assess the impact of intraoperative radiotherapy in modulating the immunological response and wound healing process. Thus, the main aim of this study was to perform an analysis of the SWF composition in two groups of breast cancer patients: those patients who underwent BCS followed by IORT treatment, and those who underwent BCS only. Clarification of the composition of SWF will allow a better understanding of the clinical observations obtained through the frequent utility of IORT treatment, which reduces the risk of local recurrence not only through direct cell killing, but also through modification of local microenvironment.

## 2. Results

### Analysis of the Composition of SWF from BCS Patients and BCS Patients after IORT Treatment

**Figure 1:**
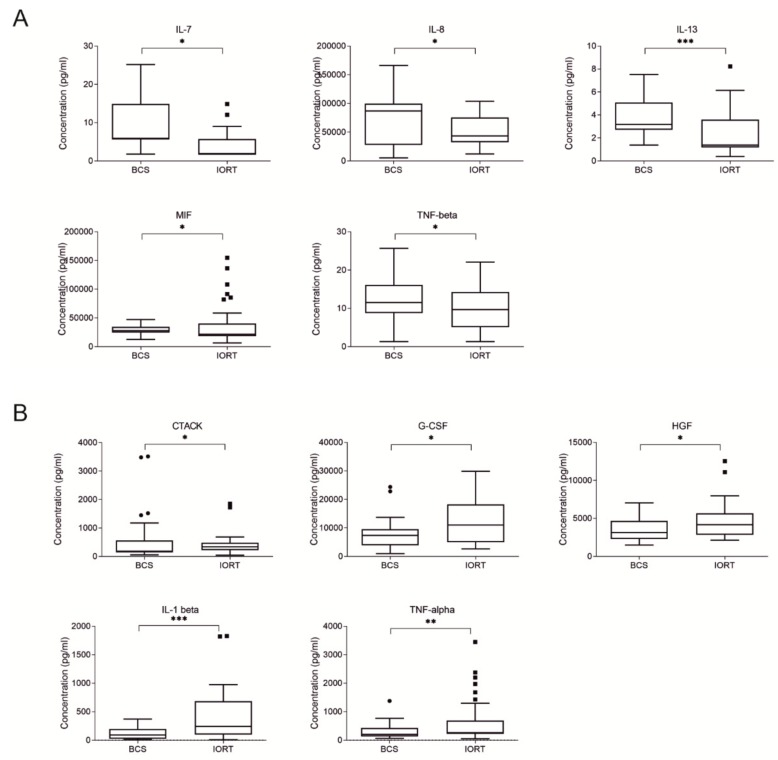
Box plots (median and whiskers) presenting concentration of cytokines (pg/ml) of surgical wound fluids (SWF) collected from patients after breast conserving surgery (BCS) and breast conserving surgery followed by intraoperative radiation therapy (IORT). (**A**) Cytokines with decreased concentrations in IORT group. (**B**) Cytokines with increased concentrations in IORT group. Whiskers were calculated using Tuckey method based on GraphPad Prism software. Outliners are shown as dots. * *p* < 0.05, ** *p* < 0.01, *** *p* < 0.001: based on Mann–Whitney test.

The main aim of this study was to perform the quantitative analysis of the SWF composition in two groups of breast cancer patients: patients which undergo BCS followed by IORT treatment, and those who underwent BCS only. Thus, surgical wound fluids from 18 BCS and 20 IORT patients were collected 48 h after the surgery. Patients classified to IORT treatment are early breast cancer patients, usually with luminal A or luminal B molecular subtype. Thus, only patients with those classification were subjected to the study. The comparison without distinguishing molecular subtype diagnosis of BCS versus IORT SWF revealed 10 cytokines which differ significantly between analyzed groups. We found that the concentration of IL-7, IL-8, IL-13, MIF (macrophage migration inhibitory factor), and TNF-beta were significantly decreased in IORT SWF compared to BCS SWF (Figure 1A). On the other hand, IORT SWF is characterized by increased concentration of CTACK, G-CSF, HGF, IL-1 beta, and TNF-alpha (Figure 1B).

**Figure 2:**
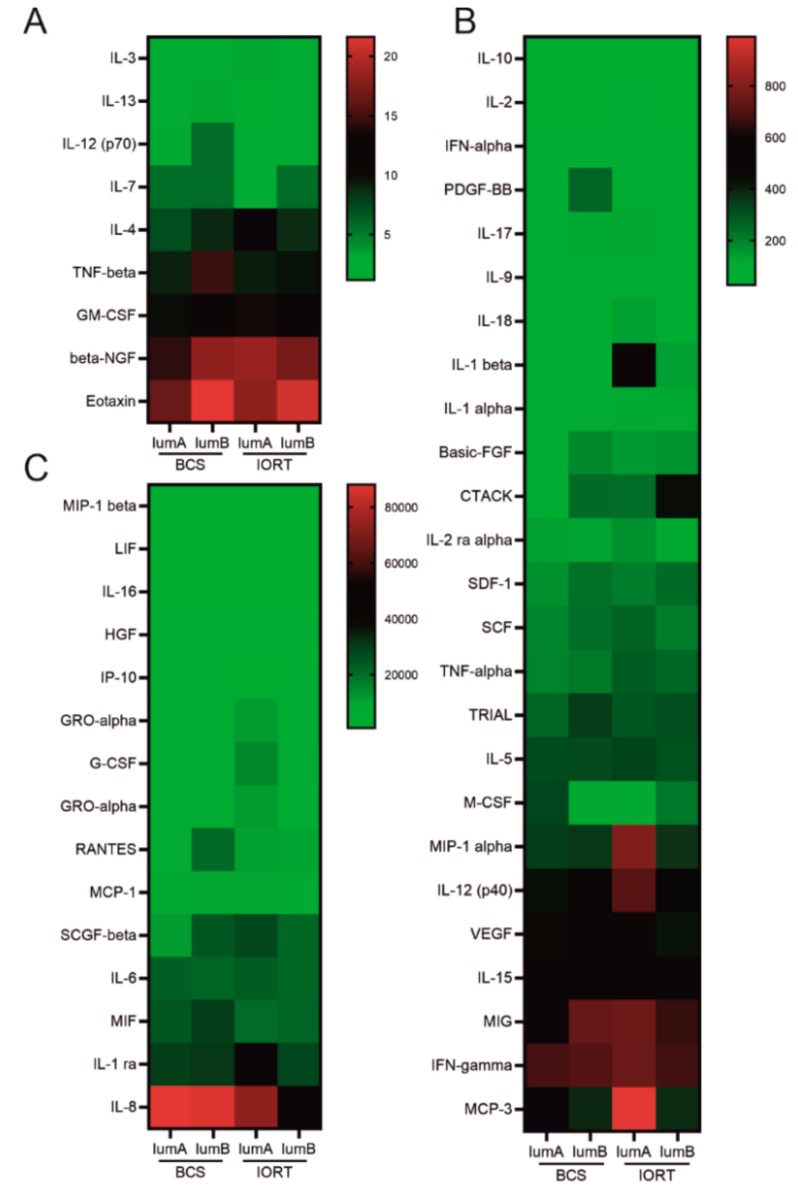
Heatmap representing concentration of all analyzed cytokines in BCS and IORT group distinguishing the molecular subtype of BC. To clarify the differences in cytokine concentrations, heatmap was divided into three: (**A**) 0–20 pg/ml, (**B**) 20–1000 pg/ml, (**C**) 1000–90000 pg/ml.

To dissect the composition of SWF according to BC molecular subtype, we divided the group of BCS and IORT SWF to luminal A and luminal B subtype (Figure 2). 

**Figure 3:**
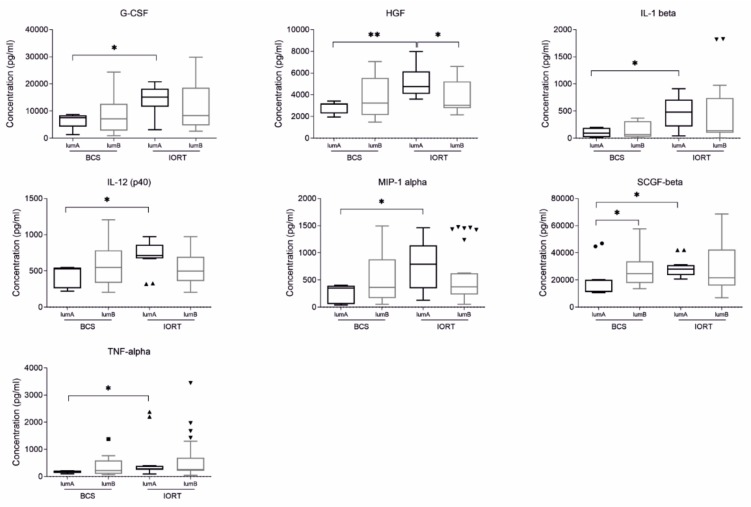
Box plots (median and whiskers) presenting concentration of cytokines (pg/ml) of surgical wound fluids (SWF) collected from patients after breast conserving surgery (BCS) and breast conserving surgery followed by IORT (IORT) in luminal A subtype of breast cancer. Whiskers were calculated using Tuckey method based on GraphPad Prism software. Outliners are shown as dots. * *p* < 0.05, ** *p* < 0.01, *** *p* < 0.001: based on Kruskal–Wallis test with Dunn’s post hoc multiple comparison test.

We found that seven cytokines were significantly changed between BCS and IORT SWF, and they were characteristic only for luminal A subtype of BC: G-CSF, HGF, IL-1 beta, IL-12 (p40), MIP-1 alpha, SCGF, and TNF-alpha (Figure 3). 

**Figure 4:**
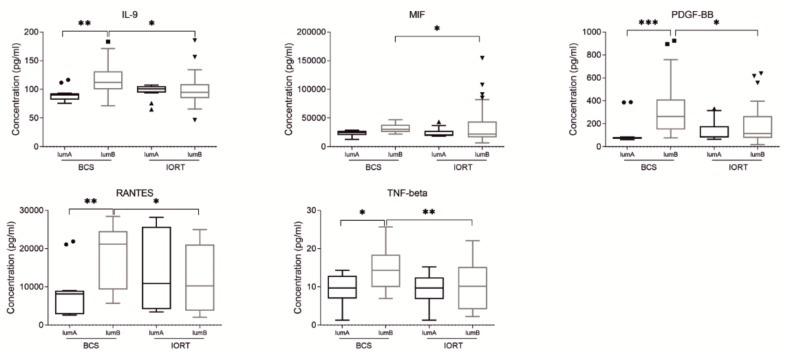
Box plots (median and whiskers) presenting concentration of cytokines (pg/ml) of surgical wound fluids (SWF) collected from patients after breast conserving surgery (BCS) and breast conserving surgery followed by IORT (IORT) in luminal B subtype of breast cancer. Whiskers were calculated using Tukey method based on GraphPad Prism software. Outliners are shown as dots. * *p* < 0.05, ** *p* < 0.01, *** *p* < 0.001: based on Kruskal–Wallis test with Dunn’s post hoc multiple comparison test.

In luminal B subtype of BC, we found five cytokines which differ significantly between BCS and IORT group: IL-9, MIF, PDGF-BB, RANTES, and TNF-beta (Figure 4).

It is worth pointing out that concentration of HGF cytokine in SWF from luminal A subtype was also significantly decreased in IORT luminal A group in comparison with IORT luminal B group (Figure 3). Moreover, we found that the concentration of SCGF (Figure 3), IL-9, PDGF-BB, RANTES, and TNF-beta, differ significantly between luminal A and luminal B BCS group.

**Figure 5:**
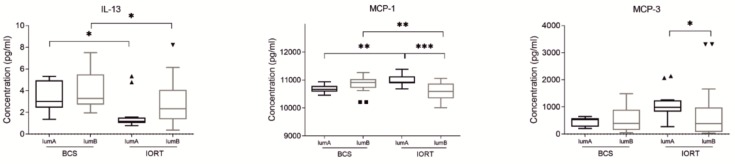
Box plots (median and whiskers) presenting concentration of cytokines (pg/ml) of surgical wound fluids (SWF) collected from patients after breast conserving surgery (BCS) and breast conserving surgery followed by IORT (IORT) in luminal A and luminal B subtype of breast cancer. Whiskers were calculated using Tukey method based on GraphPad Prism software. Outliners are shown as dots. * *p* < 0.05, ** *p* < 0.01, *** *p* < 0.001: based on Kruskal–Wallis test with Dunn’s post hoc multiple comparison test.

We found only three small molecules, which concentration differs significantly in both luminal A and luminal B subtypes of breast cancer: IL-13, MCP-1 (CCL2), and MCP-3 (CCL7). While the IL-13 concentration is significantly decreased in IORT group in both luminal A and luminal B subtype of BC, in the case of MCP-1, we observe an inverse correlation in the concentration difference between the molecular subtypes (Figure 5). In luminal A subtype, the concentration of MCP-1 is increased in the IORT group, while in luminal B subtype, its concentration is decreased in the IORT group. Moreover, a statistically significant change in MCP-1 concentration is also observed between two IORT groups (decrease in luminal B subtype). Similar differences between the IORT groups were also found in other monocytes chemotactic protein—MCP-3. Again, in IORT treated patients of luminal B subtype, the concentration of analyzed chemokine (MCP-3) in SWF is significantly decreased while comparing to luminal A subtype of BC.

## 3. Discussion

For many years it was believed, that the incidence of local recurrence and remote metastasis was much higher in patients who underwent breast-conserving surgery than those after radical mastectomy [1]. The 20-year follow up of randomized trial comparing BCS and radical mastectomy for patients with early breast cancer confirmed that BCS introduces a higher incidence of local recurrence (*p* < 0.001), although, there were no differences in the rates of contralateral-breast carcinomas, remote metastasis, or second primary cancers. Moreover, the long-term survival rate among those two groups was almost the same [16]. Thus, BCS followed by external beam radiation therapy (EBRT), delivered in fractionated doses in order to significantly reduce the risk of local recurrence [17], is now the treatment of choice for early breast cancer patients. Although the standard EBRT achieves good results, the duration of the treatment remains an obstacle. Due to breast cancer cell density (highest at up to 4 cm from the tumor edge), the probability of local recurrence is highest in the tumor bed. Therefore an additional single boost dose delivered directly to the tumor bed during the surgery, such as IORT, can significantly reduce local recurrence rates and the time of therapy comparing to standard EBRT [18,19]. This firstly is due to direct killing of residual cancer cells and also due to radiation-induced inflammation, which in turn can enhance the immunologic elimination of cancer cells. Moreover, recent studies of Meng et al. revealed that high single dose of irradiation is beneficial over fractionated irradiation due to lower activation of ATX-LPA-inflammatory cycle, which protects cancer cells from radiation induced cell death [20].

It has been suggested, that invasive procedures (such as surgery or biopsy) affect the remaining tumor cells by improving their survival and by stimulating proliferation and migration [3,4]. One of the concepts explaining this phenomenon is the induction of wound healing responses, which are responsible for tissue repair in normal tissues. However, the induction of adaptive and innate immune responses in tumor tissues stimulates cell survival and induces angiogenesis and the extravasation of circulating tumor cells. Studies of in vivo mouse models conducted by Krall et al. confirmed that the systemic inflammatory response induced after breast cancer surgery promotes the emergence of tumor cells. Moreover, the authors showed that perioperative anti-inflammatory treatment reduced the tumor regrowth, thus reducing early metastatic recurrence in breast cancer patients. This effect was associated with a release of cytokines and the mobilization of myeloid cells into the circulation of wounded mice [7]. Thus, it is believed that molecules present in SWF may affect residual cancer cells as well as modulating the immune response. It is important, therefore, to assess the impact of intraoperative radiotherapy in modulating the immunological response and wound healing processes. Thus, the main aim of this study was to carry out an analysis of the SWF composition in two groups of breast cancer patients: those patients who underwent BCS followed by IORT treatment, and those who underwent BCS alone.

To clarify the composition of SWF in BC patients, Wang et al. performed high-throughput screening of cytokines, chemokines and matrix metalloproteinases. In their analysis the authors focused on the time of SWF collection (1, 2, 3, and 4 days after the surgery), additional treatments such as neoadjuvant chemotherapy, TNM stage, and pathological type (carcinoma in situ, invasive breast cancer). They found that the highest concentration of cytokines was detected 1 day after the surgery and subsequently decreased day by day. They also found significant differences in levels of factors in SWF in relation to neoadjuvant chemotherapy, TNM stage, and the pathological type of BC [9]. In their in vitro studies, Wang et al. also confirmed the results of previous experiments [4,12], showing that SWF significantly stimulates proliferation of breast cancer cells. Similar experiments were also conducted by Agresti et al. who confirmed that the composition of SWF depends not only on the type of surgical procedure (quadrantectomy, mastectomy), but also on the aggressiveness of the tumor (in situ vs invasive), molecular subtype (luminal vs. triple-negative), the size of the tumor, staging (G2 or G3), and lymph node status (positive or negative for cancer cells). They suggested that the composition of surgical wound fluids collected from the tumor bed of breast cancer patients reflects the inflammatory nature and aggressiveness of the breast tumor [8]. The authors analyzed a panel of 34 cytokines, chemokines, and growth factors in the SWF of breast cancer patients and found, that the concentrations of IP-10, IL-6, G-CSF, osteoponin, MIP-1 alpha, MIP-1 beta, and MCP1-MCAF were higher in more aggressive tumors. 

In this study, we have demonstrated that intraoperative radiation electron therapy treatment (10 Gy, 9 MeV, Mobetron) changes the composition of surgical wound fluids collected from breast cancer patients after breast-conserving therapy. We have also shown that the composition of SWF differs between two of the molecular subtypes of BC: luminal A and luminal B. In our study, we focused on quantitative analysis of individual SWF samples collected 48 h after BCS (Table 1). 

The levels of cytokines in SWF from patients with benign breast lesions and breast cancer of different histopathological types and grades has already been investigated by Lyon et al. [38]. However, the difference between SWFs collected from cases of the luminal A, and luminal B molecular subtypes of BC have not been previously studied. An analysis comprising a comparison of the cytokines in SWF collected from IORT treated patients with those in the SWF of BCS patients has also never been performed. The approaches of the groups mentioned above differ significantly from ours, however. Firstly, the IORT patients in Belletti’s study were treated in TARGIT trial, where 50 keV photon energy was used to deliver a dose of 20 Gy to the tumor bed. Secondly, a global analysis of cytokines was performed on pooled patient samples, and finally, patients were not grouped based on histopathological type or grading. Moreover, little of the presented data was based on quantitative analysis. In the studies conducted by Belletti et al., the authors named 10 cytokines to be increased by TARGIT and 20 to be decreased by TARGIT. Among these were cytokines which were also found to differ between groups in our studies. Cytokines which were found to be reduced after IORT treatment in both Belletti’s work and ours include IL-7, MCP-1 (only in the luminal B subtype in our study), RANTES, PDGF-BB, and IL-8. Among the cytokines of increased concentration, only G-CSF was found in both studies. We found differences between several cytokines in the two studies: HGF and MIP-1 alpha were decreased in Belletti’s studies, whereas we found a significant increase in their concentration in the IORT treated group compared to BCS only, and IL-13 which was increased in TARGIT patients but significantly decreased in our study. The discrepancies of those results may be caused by the different radiation doses used, different energy, and the selection of the study group. An essential feature of our work is the fact that the SWF samples were analyzed individually. Although sample pooling can be an attractive approach in proteomic analysis, it can also introduce errors and biases for broadly heterogenic samples. As we and others confirmed, the profile of cytokines differs significantly due to grading, histopathological type, molecular subtype, and lymph node status. Those features should be taken into consideration while performing proteomic analyses on patients’ samples.

IL-8, the level of which was significantly decreased after IORT treatment, is a pro-inflammatory chemokine which plays an important role in tumor progression and metastasis in a variety of human cancers. It is also often associated with epithelial to mesenchymal transition and cancer stem cell features of breast cancer cells [39,40]. Studies of Charafe-Jauffret et al. on breast cancer cell lines [41] revealed that IL-8 treatment increased the formation of primary and secondary tumorospheres in a dose-dependent manner, as well as the cancer stem cell phenotype measured by aldehyde dehydrogenase (ALDH) activity. Moreover, IL-8 has also been implicated in the promotion of angiogenesis in endothelial cells, facilitating the development of cancer [29]. Epithelial to mesenchymal transition is associated with the increased invasive capacity of cancer cells and thus promotes tumor infiltration, growth, and metastasis. We have previously demonstrated that SWF stimulates the CSC phenotype and EMT program in breast cancer cell lines [14]. This effect was partially abolished when the cells were incubated in SWF collected from IORT treated patients. In this study, we showed a significant decrease of IL-8 after IORT treatment. Thus, our results agree with previously published papers, indicating that higher IL-8 concentrations correlate with EMT and CSC phenotype. Moreover, we have shown that intraoperative radiotherapy decreases the IL-8 concentration and thus EMT and CSC. The exact relationship between IL-8-IORT-EMT/CSC needs further study, however. It is also important to point out that, in the work of Belletti et al., IL-8 concentration was also reduced in SWF from IORT treated patients.

IL-13 is another cytokine which we confirmed to be greatly reduced in SWF collected from patients after IORT treatment (Figure 1 and Figure 5). IL-13 induces the activation of M2 macrophages. These cells are responsible for the promotion of inflammation, which induces angiogenesis and tissue remodeling [25]. M2 macrophages have also been implicated in tumor growth [42,43], and their presence has been correlated with a poor prognosis [44]. Recent data published by Little et al. showed that IL-4/IL-13 polarizes M2a macrophages and induces migration and invasion of breast cancer cells [26]. Based on those findings, we can assume that IORT treatment may be beneficial not only but direct tumor cell killing but also in changing the tumor bed microenvironment, making it less favorable for tumor recurrence due to decreased concentration of tumor-facilitating cytokines.

Haabeth et al. discovered that tumor-suppressive inflammation is regulated by a small number of cytokines, early in the immune response [22]. They pointed out that cancer-related inflammation processes cannot be seen as solely negative or positive. Based on their findings, as well as other data in the literature, they suggest that some of the well-known pro-inflammatory cytokines may either be cancer-suppressive or cancer-promoting [22,45]. In our study, we found increased concentrations of Il-12(p40), Il-1 beta and TNF-alpha in SWF from the luminal A subtype of BC in the group treated with IORT compared to BCS. IL-12 is known to stimulate Th1-dominant immunity, and thus has a strong anti-tumor activity [46]. It stimulates cytotoxic T lymphocytes and Th1 cells to produce IFN-γ, which inhibits the tumor cell cycle [45]. IL-12(p40) is the most important cytokine in the induction of Th1 cells. It has been suggested that activation of Th1 cells may be especially beneficial against cancer [23,24]. Th1 cells can induce the secretion of IL-1 beta and TNF-alpha by M1 macrophages and thus suppress the tumor. It has been proposed that IL-1 beta inhibits the proliferation of some cancers; amplifies the function of dendritic cells; stimulates the proliferation and differentiation of CD4, CD8, and NK cells; increases antibody production by lymphocytes B and finally increases the expression of adhesion molecules on vascular endothelium thus promoting the migration of leukocytes (reviewed in [22]).

The concentration of IL-1 beta is very high 24 h after surgery and decreases daily thereafter. It is one of the major cytokines present in SWF after surgery, especially in patients after neoadjuvant chemotherapy [9]. Agresti et al. also found a higher concentration of this cytokine in patients after radical mastectomy than in those after quadrantectomy [8]. 

TNF (tumor necrosis factor) has an important role in the tumor microenvironment. It is a pleiotropic cytokine which could be either pro- or anti-tumorigenic. On the one hand, it is connected to tumor promotion by stimulating survival, proliferation, migration, and angiogenesis in most cancer cells that are resistant to TNF-induced cytotoxicity [35,36,47]. However, TNF, by acting directly on cancer cells, can induce apoptosis and inhibit proliferation, processes that could be used for cancer therapy [37,48]. We have previously shown that SWF from patients treated by IORT significantly stimulates the apoptotic pathway in the luminal A subtype of breast cancer cells by activating the TNF-induced extrinsic apoptotic pathway [49]. 

In our study, we analyzed the composition of SWF not only dependent on the type of treatment (BCS or BCS followed by IORT), but also on two molecular subtypes—luminal A and luminal B—which have not been studied before in this context. The 10-year follow up of the clinical trial, “Intraoperative tumor bed boost with electrons in breast cancer”, revealed that two analyzed parameters: metastasis-free survival and overall survival in the luminal B subtype of BC are significantly lower compared to the luminal A subtype [50]. Our small molecules analysis revealed that the concentration of HGF, SCF, MCP-1, and MCP-3 differed significantly between SWF samples from luminal A and luminal B subtype patients after BCS followed by IORT. The MCP-1 and MCP-3 concentrations correlate with TNM stage of breast cancer patients, as shown by Wang et al [9]. They found that MCP-1 concentration decreases with TNM staging, the higher the lymph node status, the lower the concentration, while MCP-3 increases with TNM staging (the highest in T2). While in our studies we did not see any changes in inflammatory chemokine MCP-1 (CCL2) concentrations between BCS and the IORT group (Figure 1), more detailed analysis based on BC subtypes revealed significant differences. In the luminal A subtype, we observed a higher concentration of this chemokine in the IORT group than in the BCS group while in luminal B, the effect of IORT treatment was the opposite. As shown by Feng et al. [51], in breast cancer patients, high levels of MCP-1 correlates with increased monocyte mobilization and poor survival. Moreover, MCP-1 may also be involved in monocyte polarization to M2-cells [30]. A recent finding by Valeta-Magara et al. [39] showed that M2 macrophages are also associated with highly aggressive breast cancer—inflammatory breast cancer—which is characterized by a high percentage of cancer stem cells and increased mesenchymal features. 

## 4. Materials and Methods 

### 4.1. Surgical Wound Fluids Collection

Surgical wound fluids were collected as previously described [14]. Shortly, SWF were collected continuously for up to 48 h after BCS from BC patients. This time point was chosen as most of the drainages from patients after BCS in Greater Poland Cancer Center are usually collected at this time. Drainages are collected 24 h after BCS only if a high quantity of SWF is produced. Following tumor resection, one group of patients (denominated IORT group) underwent IORT boost (up to 10 Gy) to the tumor bed. For this procedure dedicated treatment accelerator Mobetron® from IntraOp® is used. This accelerator can produce a four treatment beams of electrons with energies 4,6,9, and 12 MeV and with dose rate about 1000 MU/min (usually 9 MeV is used). The therapeutic range is up to 4 cm tissue thickness. 10 Gy dose is delivered to the 90% isodose. The applicator (field size) is selected individually for each patient. Irradiated volume must contain tumor bed with resection margin (high-risk areas). The second group of patients did not receive IORT (BCS group). SWF were collected to drainage bottles, centrifuged for 25 min at 300 × g at 4 °C, sterile filtered and stored at −80 °C. The study was approved by the Bioethics Committee of Poznań University of Medical Sciences, study number 756/16.

### 4.2. Study Group

The study group was firstly divided into two groups based on the treatment scheme (BCS and IORT, see above). The BCS group consisted of 18 patients, and IORT—20 patients of both luminal A (ER receptor positive, PR receptor +/−, Her2−, Ki67 < 20) and luminal B subtype (ER receptor positive, PR receptor +/−, Her2−/+, Ki67 ≥ 20. We focused on these two molecular subtypes, as most of the IORT treated patients in Greater Poland Cancer Center are classified to those subtypes. None of the patients classified to the study underwent neoadjuvant therapy or had any incidence of earlier cancer. Each SWF sample was analyzed twice. For the analysis, we did not include SWF from ER−, PR−, HER+, and triple-negative subtypes of BC. Moreover, all patients were chosen based on histological subtype (all were classified as NST) and similar TNM status: T1, N0, or N1. In further statistical analysis, we classified patients from each treatment group to corresponding molecular subtype of BC: luminal A and luminal B. Histopathological and molecular classification of BC patients included in this study was done according to Fourth Edition of WHO Classification of Tumors of the Breast and current CAP/ASCO guidelines, respectively. Detailed patients’ characteristics are shown in Table 2.

### 4.3. Analysis of SWF Composition

The composition of SWF was analyzed on the Bio-Plex 200 system (BioRad Laboratories, Hercules, CA, USA). We took used Bio-Plex Pro Human Cytokine 48-plex, to analyze cytokines, chemokines, and growth factors that may be involved in the inflammation process, initiation, and progression of cancer. The benefit of this system is the quantitative and qualitative analysis of 48 small molecules in one sample. The concentrations (pg/ml) of analyzed molecules are compared to the standard curve of serial dilutions of standard molecules. The analysis was performed according to the manufacturer’s instructions. Each SWF sample was analyzed in technical duplicate. 

### 4.4. Statistical Analysis

Statistical analyses were performed using the GraphPad Prism software program, v.6 (GraphPad Software, Inc., La Jolla, CA, USA). As the data (pg/ml) did not follow Gaussian distribution, they were examined using Mann–Whitney test (comparing BCS versus IORT group) on the Kruskal–Wallis test. *p* < 0.05 was considered to indicate a statistically significant difference. The means of technical duplicates were visualized as box plots (median and whiskers) presenting concentration of cytokines (pg/ml). Whiskers were calculated using Tukey method based on GraphPad Prism software. Outliners are shown as dots. * *p* < 0.05, ** *p* < 0.01, *** *p* < 0.001: based on Mann–Whitney or Kruskal–Wallis test with Dunn’s post hoc multiple comparison test. Heatmaps displaying median concentrations of analyzed cytokines were prepared in GraphPad Prism. The cut-offs were adjusted empirically to visualize changes (if present) both at the top and bottom of the heatmap.

## 5. Conclusions

In this study, we have conducted the quantitative analysis of SWF composition in BC patients treated with BCS only, and BCS followed by IORT. While choosing the study group, we also considered the molecular subtype of breast cancer: luminal A and luminal B. The performed analysis revealed changes based not only on the applied treatment but also on the molecular subtype of BC. We found many cytokines, which are believed to have anti-tumor properties, to be highly expressed in luminal A subtype of breast cancer in IORT treated group. What is interesting, we also found significant differences in the composition of SWF collected from IORT treated patients with tumors of different molecular subtypes.

Based on those findings we can assume that IORT treatment may be beneficial in changing tumor bed microenvironment in addition to direct killing of residual cancer cells. IORT makes tumor microenvironment less favorable for tumor recurrence due to decreased concentration of tumor-facilitating cytokines, especially in luminal A subtype of BC. By correlating the long-term results of IOERT as a boost in the breast cancer [19] and results presented in this study, we can speculate, that changes in tumor microenvironment due to different cytokine concentration may have an impact on metastasis-free survival of BC patients after IORT. In relation to this, we also confirmed the decreased concentration of cytokines connected to EMT and CSC in IORT treated patients, which confirms our previously published results of in vitro experiments showing lower EMT and CSC phenotype in BC cells treated with SWF from IORT [14]. 

## Figures and Tables

**Table 1:** Role of immuno-modulatory molecules significantly changed in surgical wound fluids from BCS and IORT patients.

Molecule	Mechanism	Reference
CTACK	Regulates T cell recruitment under homeostatic and inflammatory conditions	[5]
G-CSF	Controls the maturation of neutrophils	[8]
HGF	Induces cancer cell migration and invasion, wound healing, angiogenesis	[21]
IL-1 beta	Inhibits the proliferation of some cancers, amplifies the function of dendritic cells, stimulates the proliferation and differentiation of CD4, CD8 and natural killer (NK) cells, increases antibody production by lymphocytes B, and finally increases the expression of adhesion molecules on vascular endothelium	[22]
IL-12	Induction of Th1 cells	[23,24]
IL-13	Induces the activation of M2 macrophages, polarizes M2a macrophages and induces migration and invasion of breast cancer cells	[25,26]
IL-7	Development of B cells and T cells, regulation of epithelial to mesenchymal transition (EMT) process in cancer	[27,28]
IL-8	Increased the formation of primary and secondary tumor spheres, as well as the cancer stem cell phenotype in BC	[29]
MCP-1	Increases monocyte mobilization and poor survival and is involved in monocyte polarization to M2-cells	[30]
MCP-3	Attracts monocytes, dendritic cells (DCs), and activated T lymphocytes, to invasion sites	[31]
MIF	Induces myeloid-derived suppressor cells in tumor microenvironment	[32]
PDGF-beta	Regulates metastasis and vascular remodeling in cancer	[33]
RANTES	Promotes breast cancer progression, contributes to monocyte migration into tumor site, induces angiogenesis, promotes matrix metalloproteinase expression	[34]
SCGF-beta	Previously not reported in cancer	
TNF	Pleiotropic cytokine, stimulates survival, proliferation, migration, and angiogenesis; induces apoptosis and inhibits proliferation	[35,36,37]

**Table 2:** Patients’ characteristics.

Feature	BCS Group (*n* = 18)	IORT Group (*n* = 20)
Age	61.6 ± 10.1	61.5 ± 12.2
Luminal subtype		
A	8	8
B Her2 positive	10 3	12 5
Lymph node status		
N0	11	11
N1	7	9
T1 classification		
a	0	1
b	5	6
c	13	13
